# Public Versus Academic Discourse on ChatGPT in Health Care: Mixed Methods Study

**DOI:** 10.2196/64509

**Published:** 2025-06-23

**Authors:** Patrick Baxter, Meng-Hao Li, Jiaxin Wei, Naoru Koizumi

**Affiliations:** 1 Schar School of Policy and Government, George Mason University, 3351 Fairfax Dr, Arlington, VA, 22201, United States, 1 (703) 993-8999

**Keywords:** large language models, sentiment analysis, natural language processing, structural topic modeling, social media discourse, ethics, medical, health knowledge, attitudes, practice

## Abstract

**Background:**

The rapid emergence of artificial intelligence–based large language models (LLMs) in 2022 has initiated extensive discussions within the academic community. While proponents highlight LLMs’ potential to improve writing and analytical tasks, critics caution against the ethical and cultural implications of widespread reliance on these models. Existing literature has explored various aspects of LLMs, including their integration, performance, and utility, yet there is a gap in understanding the nature of these discussions and how public perception contrasts with expert opinion in the field of public health.

**Objective:**

This study sought to explore how the general public’s views and sentiments regarding LLMs, using OpenAI’s ChatGPT as an example, differ from those of academic researchers and experts in the field, with the goal of gaining a more comprehensive understanding of the future role of LLMs in health care.

**Methods:**

We used a hybrid sentiment analysis approach, integrating the Syuzhet package in R (R Core Team) with GPT-3.5, achieving an 84% accuracy rate in sentiment classification. Also, structural topic modeling was applied to identify and analyze 8 key discussion topics, capturing both optimistic and critical perspectives on LLMs.

**Results:**

Findings revealed a predominantly positive sentiment toward LLM integration in health care, particularly in areas such as patient care and clinical decision-making. However, concerns were raised regarding their suitability for mental health support and patient communication, highlighting potential limitations and ethical challenges.

**Conclusions:**

This study underscores the transformative potential of LLMs in public health while emphasizing the need to address ethical and practical concerns. By comparing public discourse with academic perspectives, our findings contribute to the ongoing scholarly debate on the opportunities and risks associated with LLM adoption in health care.

## Introduction

Artificial Intelligence (AI)-based large language models (LLMs) have sparked extensive discussions within the academic community since their 2022 emergence. The rhetoric is multifaceted, with rapt users touting the highly sophisticated chatbots’ potential to assist in writing tasks. Critics caution, however, that the cultural and ethical ramifications associated with such reliance on LLMs may be a burden too costly to bear. Thus far, literature assessing ramifications of LLMs spans multiple fields, including finance [1], education [2], software programming [3], public health [4], and environmental studies [5]. These studies often focus on overlapping themes, that is, appropriate integration of LLMs, their analytical performance, and practical benefits for users. What seems to be missing is an examination of the nature of such deliberations. LLMs’ societal impact ultimately relies on these users’ verdicts, as warring technophile and luddite factions set the stage for successful technological adoption. To this end, our study assessed public opinion and perception regarding the most popular LLM widely available: OpenAI’s ChatGPT.

This study specifically focused on health care, analyzing tweets that discussed the impact of ChatGPT on the health care sector since its inception and examined how ChatGPT interacts with various public health domains, including health care management, public health, digital health, clinical medicine, and nursing science [6-8]. We reviewed the opinions and sentiment shared on X (formerly known as Twitter) in order to answer the key question: Where does public and expert sentiment lie on ChatGPT’s use in health care? Health care as LLMs technology has drawn recent accolade for the potential use, in part due to ChatGPT’s recent accomplishment of passing the US Medical Licensing Exam [9]. Key concerns raised by the scientific community thus far include consequences of potential biases introduced in the LLM algorithm training process, which may exacerbate the existing health disparities [10], spreading misinformation [11], and medical record breaches and cyber security [12]. In our current work, we aimed to understand how public opinions and sentiment about LLMs contrast with the opinions shared among academic researchers and other field experts to gain a broader view for the future direction of LLMs in health care. Through opinion mining, we identified 8 topics that represent general concerns and optimisms toward ChatGPT in health care. For the analysis and classification of twitters sharing positive and negative sentiments, we implemented 4 algorithms, determining their accuracy based upon a manual review of the tweets themselves conducted by our research team. We find our novel enhanced method that combines Syuzhet and GPT 3.5 had an 84% accuracy rate, 12 percentage points better than other classification algorithms used in our analysis.

The remaining article is ordered as follows: first, we state our research goal and key question underlying our analysis. Next, we state out our statistical methods. We then present our findings and discuss the implications for future research.

## Methods

### Ethical Considerations

To protect the privacy and confidentiality of study data, all IDs, usernames, and tweets have been deidentified. Additionally, any full tweets cited in the paper have been paraphrased to prevent them from being traced back to the original user.

### Data Source

We used the academic Twitter API to retrieve tweets with search terms “ChatGPT AND (health OR healthcare OR hospital OR physician OR nurse OR nursing OR patient)” [13,14]. This data collection process was executed for the period between December 1, 2022, the day after ChatGPT became publicly available, and March 20, 2023. After removing duplicates using the Jaccard Similarity score [15], there were 6138 unique tweets authored by 4837 distinct accounts. The Jaccard Similarity is expressed as:


$$J(A,B)=\frac{|A\cap B|}{|A\cup B|}$$

where A and B are 2 sets, ∣A∩B∣ represents the number of common words between them, and ∣A∪B∣ represents the total number of unique words across both sets. The Jaccard score ranges from 0 to 1, where 0 indicates no similarity and 1 indicates identical sets.

It is worth noting that the academic Twitter API can be used to collect all tweets instead of just sampled tweets. Academic researchers are granted special access to the Twitter V2 API, which provides access to X’s real-time and all historical public data (unbiased tweets). This API is no longer supported by X Corporation. However, users can still obtain full access to X through a paid subscription.

### Sentiment Classification and Analysis Procedure

Our analysis consisted of three phases: (1) human-labeled sentiment tweet classification, (2) algorithm-based sentiment tweet classification; and (3) structural topic modeling (STM) to distinctly group tweet content. Each phase is detailed below.

#### Human-Labeled Sentiment Classification

A team of 2 public health faculty members and two PhD students first reviewed each tweet and classified them into 3 mutually exclusive sentiment categories: positive, negative, and neutral. The team categorized tweet sentiment based on lexical content, context, emojis (eg, 😊 for positive, 😡 for negative), and tone. Positive tweets typically use words like “happy” or “love,” while negative tweets include “terrible” or “hate”; neutral tweets lack strong emotions, informative contexts or advertisements. Each tweet was reviewed by 2 reviewers. If the 2 reviewers disagreed, the team discussed how to label the tweet in order to reach a consensus. Of the 6138 tweets, the majority of tweets were classified as neutral sentiment (4359/6138, 71%), while only a small percentage of tweets were classified as positive (1350/6138, 22%) and negative sentiments (460/6138, 7.5%). However, sentiment analysis models typically struggle with neutral context. Most of the collected tweets with neutral sentiment were those that included both positive and negative sentiments, potentially leading to misinterpretation of the results. Due to the inherent challenges associated with accurately interpreting neutral tweets, we excluded them from the analysis [16]. After removing neutral sentiment tweets, our final dataset contained 1806 tweets authored by 1586 distinct accounts.

#### Algorithm-Based Sentiment Classification

Next, we compared sentiment classification between 3 ML algorithms and those derived from the research team’s manual review. These ML algorithms consisted of 2 of OpenAI’s API models, Gpt-3.5-Turbo-0301 and Gpt-4.0, and the conventional dictionary-based Syuzhet method [17]. Both GPTs and Syuzhet can be used for sentiment classification, although they serve distinct purposes within this domain. GPT, generating text based on extensive pretraining text data, has a better understanding of context and is suitable for various tasks. In contrast, Syuzhet is specifically designed for sentiment analysis tasks and provides predicted labels based on its training on sentiment-labeled data. We used the Syuzhet package in R to evaluate the textual content extracted from the X data, tokenize the input text into words, then mapping them to predefined sentiment scores based on the chosen lexicon. For example, Syuzhet assigns a positive word (eg, love or happy) a+1and a negative word (eg, bad or terrible) a −1 and then adds them together for the sentence.

We explored the potential for enhancing the performance of algorithms by combining predictions from the Syuzhet and OpenAI GPT algorithms. Here, we identified the GPT algorithm with the superior performance, either GPT 3.5 or GPT 4.0, and then compared the GPT predictions with the Syuzhet predictions. If both the GPT and Syuzhet predictions exhibit the same direction, the predictions are retained and compared with the ground truth values. Each true match (ie, a tweet with the same algorithm and human labeling result) was considered a success, while contradictory results were considered an inaccurate algorithm classification. The accuracy rate was calculated as:


$$Accuracy\;rate=\frac{True\;Positive+True\;Negative}{True\;Positive+True\;Negative+False\;Positive+False\;Negative}$$

#### Application of STM

We used a STM to classify the tweets into distinct content topics discussing ChatGPT [18]. STM is a natural language processing and text analysis statistical technique that builds upon traditional topic modeling approaches, such as Latent Dirichlet Allocation [19]. Rather than simply identifying content topics, STM analyzes both the overall structure of the documents (eg, the arrangement of sentences, paragraphs, and other textual components) as well as the associations with other metadata (covariates). The approach allows for a more nuanced understanding of the topics, as well as how the topics relate to each other and to other contextual covariates. In this study, we incorporated 2 specific covariates, namely our human-labeled sentiment (positive and negative) and opinion leader status. The opinion leader covariate is defined by the number of followers associated with a given X account [20,21]. We categorize an “opinion leader” as an account in the top 10% (159/1586) of followed accounts, requiring at least 10,048 followers.

**Figure 1.**
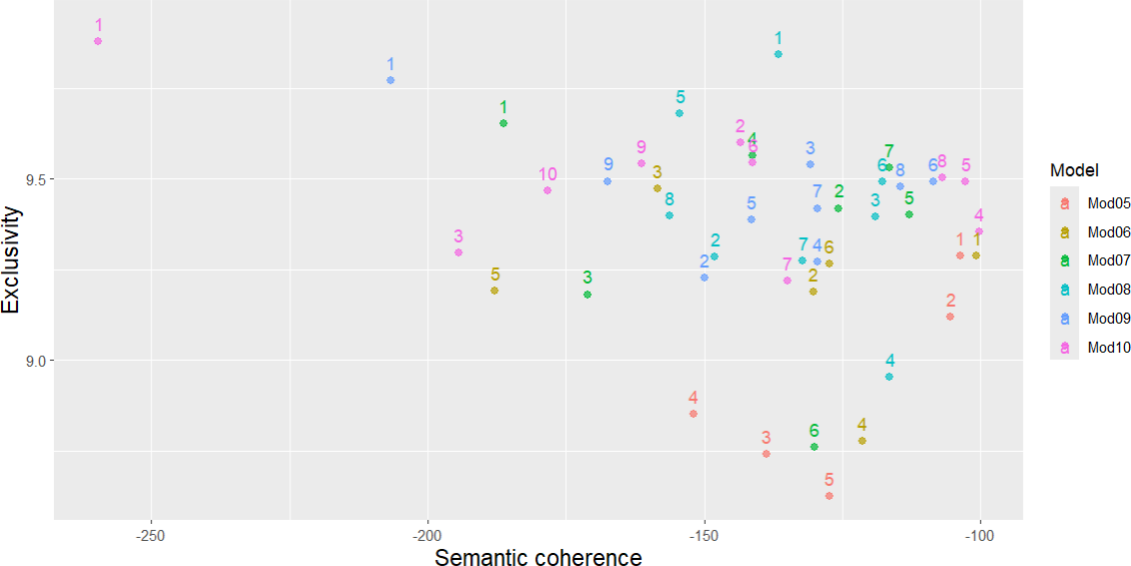
Comparison between exclusivity and semantic coherence.

The STM method itself was an elaborative process. Initially, researchers arbitrarily determined the appropriate number of topics to batch the corpus content. We experimented with 5 to 10 topics (Figure 1). Once the number of topics was established, the STM algorithm proceeded to randomly assign topics to each word in every document within the corpus. The algorithm then refined these initial assignments iteratively by analyzing both the frequency of each topic’s appearance in a document and the distribution of words within each topic. This dynamic process resulted in ongoing adjustments to the topic assignments for each tweet, while taking the probabilities of topic occurrences and word distributions into consideration. Two key measures determined the optimal number of STM topics: semantic coherence and exclusivity. Semantic coherence assesses how frequently words co-occur within a topic, reflecting the strength of their thematic connection. Topics with high semantic coherence contained words that often appear together, indicating a strong thematic link. Exclusivity quantified the distinctiveness of words within a topic, indicating how unique they are to that topic compared to others. Topics with high exclusivity contained words that are specific to that topic and rarely appear in other topics [18].

After finalizing the topics, we evaluated each topic closely in order to assign a specific label to the topic. Further, we descriptively compared the opinion leaders’ and nonopinion leaders’ tweets.

## Results

### Comparison of Sentiment Classification Algorithms

Table 1 presents the results of sentiment analyses performed using: (1) human labeling; (2) Syuzhet; (3) OpenAI GPT 3.5; and (4) GPT 4.0. True positive and true negative cases are presented in bold in the table. As seen in the table, the model performed far better in predicting positive cases with the accuracy rates of 47% (Syuzhet) and 55% (OpenAI GPT 3.5 and 4.0). For the negative cases, OpenAI GPT 3.5 performed far better than the other algorithms with the accuracy rates of 9% (Syuzhet), 18% (OpenAI GPT 3.5) and 10% (OpenAI GPT 4.0). Consequently, for the overall accuracy, OpenAI GPT 3.5 outperformed GPT 4.0 and Syuzhet with the accuracy rates of 72.04%, 65.23%, and 55.82% respectively.

**Table 1.** Human labelling versus sentiment classification algorithms.

	Syuzhet			GPT3.5			GPT4.0		
	Negative	Positive	Neutral	Negative	Positive	Neutral	Negative	Positive	Neutral
Ground Truth									
Negative, n (%)	163 (9.03)^a^	165 (9.14)^b^	135 (7.48)^c^	316 (17.50)^a^	17 (0.94)^b^	130 (7.20)^c^	178 (9.86)^a^	15 (0.83)^b^	270 (14.95)^c^
Positive, n (%)	73 (4.04)^c^	845 (46.79)^d^	425 (23.53)^c^	43 (2.38)^c^	985 (54.54)^d^	315 (17.44)^c^	20 (1.11)^c^	1000 (55.37)^d^	323 (17.88)^c^

a True negative.

b False positive.

c False negative.

d True positive.

Table 2 presents the result of the algorithm that combined Syuzhet and OpenAI GPT 3.5. The enhanced ML approach yielded a superior accuracy rate of 84.04%, surpassing the accuracy of any single algorithm mentioned above by at least a 12% improvement rate.

**Table 2.** Human labelling versus. (Syuzhet+ OpenAI GPT 3.5)^a^.

	Syuzhet + OpenAI GPT 3.5		
	Negative	Positive	Neutral
Ground truth			
Negative, n (%)	111 (12.74)^a^	6 (0.69)^b^	34 (3.9)^c^
Positive, n (%)	11 (1.26)^c^	621 (71.3)^d^	88 (10.1)^c^

a True negative.

b False positive.

c False negative.

d True positive.

### Evaluation of Structural Topic Model Results

Figure 1 demonstrates how these topics were assessed using semantic coherence (x-axis) and exclusivity (y-axis). This evaluation guided our decision in selecting the most appropriate models for clustering the tweets. Topics positioned near the upper right corner represent higher semantic coherence and exclusivity. The evaluation indicates the “Mod08” model, which consists of 8 topics, demonstrates both high semantic coherence and high exclusivity. In other words, Mod08 best embodies a strong thematic grouping with distinctive and closely related words compared to other STMs. The corpus was therefore divided into 8 topics for the subsequent analysis.

Table 3 lists the top 20 words with the highest probabilities that appear in the tweets classified under each of the eight topics. While most tweets focused on ChatGPT’s utility in the health arena, some tweets referred to the utility of ChatGPT in multiple sectors or industries, including the health sector. These tweets were classified under Topic 1. The tweets under Topic 2 predominantly focused on the potential and plausible future roles of ChatGPT in our daily lives with significant optimism. Under Topic 3, there were a number of tweets referring to the limitations of ChatGPT due to its sole reliance on data in making decisions. The tweets under Topic 4 referred to the utility of ChatGPT as an existing mental health service provider. Tweets classified under Topics 5 and 6 focused on plausible near future transformation in health care system (Topic 5) and services (Topic 6) triggered by ChatGPT. Topic 7 tweets also commented on future roles of ChatGPT in healthcare services, but the discussions were more on the concerns stemming from biases and inaccurate information identified in ChatGPT’s responses. Finally, Topic 8 contained tweets expressing positive sentiment about ChatGPT’s capability in improving clinical documentation’s effectiveness and precision as well as its availability to respond to medical questions around the clock, many of which referred to the possibility of physicians and other healthcare professionals being replaced in the near future.

**Table 3.** Top 20 words with the highest probabilities of each topic.

Topic	Top 20 words
Topic 1	health care, industries, industry, revolutionize, finance, applications, customer, revolutionizing, exciting, impact, possibilities, various, healthtech, development, efficiency, efficient, forbes, save, lets, delivery
Topic 2	potential, technology, improve, education, future, like, care, openai, artificialintelligence, new, see, ways, way, health, medicine, innovation, service, outcomes, world, work
Topic 3	time, tool, data, one, like, human, better, see, much, hospital, able, read, responses, day, school, never, imagine, dont, far, next
Topic 4	health, mental, can, support, using, advice, issues, used, people, chatbot, mentalhealth, users, public, provide, app, openai, chat, gpt, company, ethical
Topic 5	will, use, just, think, great, going, well, writing, already, take, amazing, change, cases, example, interesting, things, tech, field, many, cant
Topic 6	can, medical, patient, used, patients, language, treatment, tools, diagnosis, professionals, clinical, chatbots, intelligence, provide, help, artificial, models, accurate, data, large
Topic 7	can, help, even, care, get, people, may, information, doctor/s, patient, better, system, made, make, replace, way, want, wrong, like
Topic 8	asked, write, google, questions, patient, good, physician, answer, ask, still, using, lot, work, now, medical, best, doctors, say, need, asking

We reviewed all of the tweets under each of the topics and used these words as a supplement to label the eight topics as mentioned in Textbox 1:

Textbox 1. **Eight topics.**

- Topic 1: ChatGPT’s potential in advancing various industries
- Topic 2: ChatGPT’s potential in improving our daily lives
- Topic 3: Concerns related to ChatGPT’s reliance on data
- Topic 4: ChatGPT in mental health services
- Topic 5: ChatGPT as text generator
- Topic 6: ChatGPT as an analytical tool
- Topic 7: Fairness in ChatGPT responses
- Topic 8: ChatGPT’s potential in replacing healthcare professionals

### Representative Tweets

We summarized each of the 8 topics identified via opinion mining and provided several representative tweets under each topic to demonstrate key points and discussions surrounding each topic.

#### Topic 1: ChatGPT’s Potential in Advancing Various Industries

The 49 tweets under Topic 1 highlighted ChatGPT’s potential to trigger significant transformations across various sectors, such as retail, health care, and entertainment, through real-time applications. Approximately 90% (44/49) of these tweets expressed a positive outlook. These tweets provided concrete examples of how these industries could benefit from advancements in AI and state-of-the-art technologies.

Across sectors like retail, healthcare, and entertainment, visual ChatGPT offers a range of real-time applications poised to transform entire industries.

With advances in AI and other cutting-edge technologies, ChatGPT is steadily gaining traction in the market. Its integration into the healthcare industry? Absolutely possible.

The impact of #ChatGPT across industries is already unfolding—from healthcare to finance, its potential is immense. As conversational AI continues to evolve, it’ll be exciting to see what the future brings.

#### Topic 2: ChatGPT’s Potential in Improving Our Daily Lives

Topic 2 contained 355 tweets, with about 97% (344/355) expressing a positive sentiment. These tweets highlighted the role of innovative technologies, such as ChatGPT, in improving various aspects of our lives, including health care and transportation. Well known figures, such as Bill Gates, have emphasized ChatGPT ’s significance in revolutionizing office operations, health care, and education for better outcomes and efficiency. ChatGPT is seen as a pivotal innovation with the potential to reshape diverse domains and create numerous opportunities for innovation ecosystems. In summary, these tweets emphasize the transformative impact of AI technologies, particularly ChatGPT, in enhancing our daily lives and addressing pressing challenges, underscoring the opportunities they offer for innovation ecosystems.

It’s exciting to see how #AI is enhancing our lives across the board—from healthcare to transportation. With technology on our side, the future is looking brighter than ever.

Bill Gates believes AI—especially tools like #ChatGPT—is currently the “most important” innovation. AI technology offers powerful opportunities to boost efficiency and outcomes in workplaces, healthcare systems, and educational settings.

In conclusion, ChatGPT is as a versatile AI tool with the potential to significantly improve many areas of our lives—from personal productivity and education to health, career growth, financial planning, customer service, and even virtual events.

#### Topic 3: Concerns Related to ChatGPT’s Reliance on Data

The 136 tweets under Topic 3 placed an emphasis on concerns related to the utilization of ChatGPT in health care applications. Approximately 43% (58/136) of these tweets conveyed a negative sentiment. A primary concern pertained to the feasibility of integrating ChatGPT into health care scenarios with limited patient data and the fiscal constraints imposed by insurance companies, thus underscoring the financial considerations associated with data acquisition. Another worry centered around the security of sensitive medical data, and risks associated with disclosing such information on public domain. In addition, doubts were expressed regarding ChatGPT’s ability to offer appropriate medical advice. In summary, these tweets collectively highlighted concerns about the adoption of ChatGPT in health care services and advocated for a more cautious approach in handling sensitive medical data.

Feeding ChatGPT data equations, flowcharts, and calculations? That’s the easy part. Now put it in a patient room—with a poor historian and limited information—and expect a clear answer? Good luck. Oh, and by the way—insurance doesn’t always approve more tests. Data isn’t free.

Y’all, what are you doing? Treat any data you give to OpenAI like it’s going on your public Facebook feed. Would you post patient info on FB? Then don’t put it in ChatGPT. Would you share your company’s financials on FB? Then don’t feed them to ChatGPT either. Come on—this is rookie league stuff.

I’ve been steering clear of (chat)gpt’s hype—and turns out, I was right. Don’t rely on ChatGPT for health advice. Tools like https://t.co/z0nixzpowi are far better for lit reviews and won’t mislead you with false hope or questionable treatments.

#### Topic 4: ChatGPT in Mental Health Services

Topic 4 encompassed 387 tweets, with approximately 52% (201/387) of them conveying a negative sentiment. These tweets shed light on the debates surrounding the utilization of ChatGPT in mental health applications. Supporters argued that ChatGPT could offer a valuable alternative to traditional therapy for individuals who face financial constraints or prefer remote interactions, thereby enhancing the accessibility of mental health services. Furthermore, health apps using ChatGPT as an AI health coach can provide personalized and round-the-clock assistance, potentially revolutionizing the field of health coaching. However, ethical concerns were raised, particularly in terms of informed consent. Some view experiments like Koko’s use of ChatGPT for mental health support as ethically questionable. In conclusion, although ChatGPT shows promise in addressing mental health needs, it is imperative to carefully navigate ethical considerations and consent issues to ensure its responsible implementation in this domain.

ChatGPT has the potential to serve as a digital therapist for those who can’t afford counseling or prefer to avoid in-person sessions. It could help expand access to mental health support for people who need it the most.

A health app now uses ChatGPT to take the place of human health coaches—giving users round-the-clock access to an AI coach that offers clear, helpful support and advice based on their needs.

I honestly don’t see how an experiment like this could be exempt from informed consent requirements. It’s flat-out #unethical. A company using #ChatGPT for mental health support without proper safeguards brings serious ethical concerns to the table.

Here we go—Koko, a nonprofit focused on peer mental health support, ran a test using ChatGPT on its users without getting their consent. That’s a serious breach of trust.

#### Topic 5: ChatGPT as Text Generator

Topic 5 contained a total of 169 tweets, with the majority (132/161, 82%) reflecting a positive sentiment. These tweets highlight the growing recognition of ChatGPT’s remarkable applications in various domains, particularly for remote health care delivery and summarizing virtual meetings. Its automation capabilities and ability to provide insightful summaries generated positive feedback from users, suggesting its potential to disrupt conventional industries such as consulting and academia. In short, ChatGPT is demonstrating its transformative potential across multiple domains, reshaping our approaches to writing, summarizing, and decision-making.

At our organization, we’re currently experimenting with ChatGPT in the healthcare space—especially in delivering remote care globally. It’s been helpful for generating automated summaries and transcripts of virtual sessions. At this point, its usefulness is hard to deny.

The tools coming out of OpenAI are already shaking up the overpriced consulting world—and honestly, good! Just yesterday, a friend told me their buddy, who's on the hunt for a nursing job, was amazed at how much ChatGPT helped them craft a strong resume.

Truly impressive—I asked ChatGPT how the #healthcare system might evolve after COVID-19, and it delivered a thoughtful response in just 3–5 seconds. It’s clear that #academia needs to start engaging with this tool in a smart, thoughtful way.

#### Topic 6: ChatGPT as an Analytical Tool

Topic 6 contained a total of 288 tweets, with the vast majority (268/288, 93%) conveying a positive sentiment. These tweets emphasized the crucial role of ChatGPT in health care decision-making and highlighted several key aspects. ChatGPT assists physicians with analyzing patient symptoms and medical history, facilitating diagnoses, and personalized treatment recommendations, and helping physicians make more informed decisions. Finally, ChatGPT serves as a valuable resource for lay people to understand medical conditions, drug interactions and treatment options, supporting decisions based on individual needs.

One way ChatGPT can support the medical field is by helping professionals consider possible diagnoses and treatment paths. By reviewing patient symptoms and medical history, it can suggest options that aid doctors in making more informed choices.

ChatGPT can support healthcare by delivering fast, accurate responses to medical questions, aiding in clinical decision-making, and offering suggestions that reflect each patient’s individual needs and situation.

ChatGPT can assist doctors by offering information on medical conditions, potential drug interactions, and available treatment options—helping support their work in diagnosing and treating patients.

Conversational AI can play a role in telemedicine by helping patients reach healthcare professionals and offering useful information about their health along the way.

ChatGPT could serve as a source of reliable, up-to-date information on a wide variety of health topics—from common illnesses to available treatments and therapies.

#### Topic 7: Fairness in ChatGPT Responses

There were 176 tweets categorized under Topic 7. Approximately 35% (62/176) of the tweets expressed negative sentiments. The negative tweets highlighted concerns about biases seen in ChatGPT’s responses in health-related conversations. When requesting stories involving doctors and nurses, the AI often portrays nurses as women and doctors as men. Similarly, some professions are likely to be portrayed with a specific sex and performance reviews generated by ChatGPT tend to be more critical for female employees, exhibiting possible gender as well as role biases. In addition, some tweets acknowledged that small errors generated by ChatGPT could potentially cause serious harm to patients.

I asked ChatGPT for ten stories involving a doctor and a nurse. Only one featured a female doctor and a male nurse. Every story had a heterosexual pairing, and the names were overwhelmingly Anglophone—doctors named Alex, Jack, and Rachel; nurses with similar naming patterns. AI is still echoing bias, not representing reality.

AI often mirrors the biases in its training data. Ask for a picture of a nurse, and you’ll probably see a woman. Ask for a doctor, and chances are you’ll get a man. This isn’t just coincidence—it’s a reflection of long-standing stereotypes baked into the data.

ChatGPT tends to write longer and more critical performance reviews when it assumes the employee is a woman. It associates roles like nurse, receptionist, and kindergarten teacher with women, while seeing mechanic as male, and banker or engineer as male or neutral. These patterns show how gender bias can still surface in AI-generated content.

The issue with ChatGPT is that it can still make mistakes—even in basic essays, including historical or factual ones. Sure, it might get 95% of the information right, but that remaining 5%? In medicine, that margin of error could cost a life. We’re still years away from trusting chatbots in the operating room.

#### Topic 8: ChatGPT’s Potential in Replacing Healthcare Professionals

Topic 8 consisted of 266 tweets, and 29% (77/266) of them expressed negative sentiments. These tweets offered a glimpse into the various perspectives on ChatGPT’s potential in substituting health care professionals. Some individuals believed that ChatGPT has demonstrated promising prospects in the health care arena and has the ability to generate text of human-like quality. For example, ChatGPT could transcribe patient audio and produce medical correspondence, possibly improving clinical documentation’s effectiveness and precision. However, it is critical to acknowledge that ChatGPT is not a substitute for human expertise at this point. While ChatGPT can offer valuable assistance, particularly in generating high-quality human-like text, its performance in certain areas, such as health care real estate, is still inadequate. This implies that more refinement and development is required before it can completely replace human expertise in every aspect of health care.

Using OpenAI’s open-source Whisper to transcribe patient audio, then running it through ChatGPT for responses—it’s a setup that could be built at low cost and, frankly, might outperform your average BetterHelp therapist.

I just fed some brief (fictional) patient notes into ChatGPT and asked it to draft a medical letter—the outcome was surprisingly solid. Honestly, it’s getting close to dictation-level quality.

ChatGPT is out here solving problems we didn’t really have. What I actually need is a system that makes healthcare affordable. Or a robot that can clean my place. Not a faster version of Google.

I ran some detailed healthcare real estate questions by ChatGPT—stuff I already knew the answers to—and it came back with a vague, off-the-mark response. Safe to say, my job’s not going anywhere anytime soon.

### Comparison Between Public and Opinion Leaders

Table 4 presents the number and percentage of tweets by opinion leader status. Overall, both groups exhibited similar tweeting patterns. Opinion leaders were, however, more likely to discuss: (1) ChatGPT’s potential in improving our daily lives (Topic 2: 22.48% vs 18.01%); and (2) the possibility of ChatGPT replacing health care professionals (Topic 8: 20.18% vs 13.98%). In contrast, nonopinion leaders expressed more concern about the use of ChatGPT as an analytical tool in their daily lives (Topic 6:16.75% vs 10.09%). These findings illustrated the diverse perspectives and priorities within the ChatGPT discourse, underscoring the importance of considering multiple viewpoints when evaluating its impact on industries and daily lives.

**Table 4.** Comparison between opinion leaders’ and nonopinion leaders’ tweets.

	Opinion leader, n (%)	Nonopinion leader, n (%)
Topic 1: ChatGPT’s potential in advancing various industries	2 (0.92)	47 (2.96)
Topic 2: ChatGPT’s potential in improving our daily lives	49 (22.48)	286 (18.01)
Topic 3: Concerns related to ChatGPT’s reliance on data	19 (8.72)	117 (7.37)
Topic 4: ChatGPT in mental health services	46 (21.1)	341 (21.47)
Topic 5: ChatGPT as text generator	16 (7.34)	153 (9.63)
Topic 6: ChatGPT as an analytical tool	22 (10.09)	266 (16.75)
Topic 7: Fairness in ChatGPT responses	20 (9.17)	156 (9.82)
Topic 8: ChatGPT’s potential in replacing healthcare professionals	44 (20.18)	222 (13.98)

## Discussion

### Principal Findings

This study performed public sentiment analysis regarding ChatGPT’s impact on health care. The findings provide several implications for both the deployment of LLMs in healthcare and the need for broader understanding of public opinion towards AI technologies in medical contexts.

The predominance of positive sentiment toward ChatGPT indicates a general optimism amongst X or Twitter users about the integration of AI into the field. This optimism was notably strong in discussions surrounding ChatGPT’s potential to enhance patient care and health care decision-making, perhaps an acknowledgment of AI’s capacity to process and synthesize large amounts of medical information quickly and accurately, which can support medical professionals in diagnosing and treating patients more effectively. In addition, users were excited about ChatGPT’s availability to respond to questions.

Conversely, the areas of concern highlighted by the negative sentiments—primarily around mental health support and patient communication—point to critical ethical and practical challenges. Concerns of the reliability of AI-generated advice, the management of patient data, and the potential for perpetuating biases within AI algorithms are prevalent across topics. This suggests while there is readiness to embrace AI for certain technical tasks within health care, cautious public concern remains regarding the limits of AI’s role in sensitive aspects of health care. Empathetic human interaction can and should play a crucial role in these areas.

The added sentiment classification accuracy of the enhanced ML approach is also worthy of note. Combining Syuzhet and OpenAI GPT 3.5 algorithm predictions resulted in an 84% accuracy rate, by far the best performing classification strategy we tested for our analysis. We theorized that by integrating the Syuzhet approach, which focuses on extracting the underlying emotional trajectory of a narrative with the predictive power of OpenAI’s GPT 3.5. The model’s synergy allowed for a more robust interpretation of data. Future researchers should iterate our approach with more sophisticated LLM models, like ChatGPT 4.0 or ChatGPT 4.5, to further enhance accurate sentiment analysis. The high accuracy rate implies that using both the GPT models and the Syuzhet package, researchers or policy makers can efficiently monitor public sentiment on emerging health crises and quickly analyze public health sentiment and meaningful insights on Twitter (or other social media) without extensive expertise in natural language processing. Also, recent studies on sentiment analysis of ChatGPT tweets typically use machine learning approaches for classification and require researchers to manually label tweets to create a training set [22-24]. The human labelling process is labor-intensive and time-consuming. In our study, we demonstrated that pretrained LLMs are a potential tool to classify tweet sentiment without the need for manual labeling, significantly reducing the time and effort required by researchers.

Overall, public sentiments towards AI adoption in health care mirrored opinions found in academic literature. In particular, an abundance of literature has been published on ChatGPT’s utility as a text generator (Topic 5). Here, a number of academic publications focus on the use of ChatGPT in scientific publications, which has led many journals and publishers to set new restrictions on the use of ChatGPT in generating manuscripts [25-28]. The main rationales for such restrictions are ChatGPT’s “artificial hallucination”, particularly in generating references that do not exist [29] as well as potential plagiarism for which non-human authors cannot be held accountable [30]. On the positive side, ChatGPT’s usefulness as a generator of patient clinical notes and discharge summaries has also been explored and discussed by academics and health professionals [31-33] which corresponds to many tweets found under Topic 5. Racial or ethnic and gender biases as well as misinformation in ChatGPT’s responses (Topic 7) are also noted in academic literature [34,35], and there is a general consensus in the literature that the challenge is likely to persist despite the use of more training data and novel algorithms [36].

Both academic literature and public tweets commented on the overconfidence of ChatGPT’s responses in providing misinformation. Topic 6 (ChatGPT as an Analytical Tool) has also been widely discussed in academic and health professional communities as a potential diagnostic tool and a recommender and a potential decision maker of treatment regimens [37-39]. These studies conclude that ChatGPT’s responses are mostly accurate on common, nonspecialized, topics, while, for specialized topics, the accuracy remains subpar. This aspect was not discussed in the tweets from the general public. Discussions about the negative consequences of limited training data (Topic 3) were seen ubiquitously as the major limitation of the currently available LLM in academic literature [36]. And the literature often perceived this issue as a long-term challenge. The literature unanimously states that health care workers cannot be replaced by AI (Topic 8), highlighting the importance of human-AI collaboration [40-42], while the general public was more likely to emphasize that human replacement is likely in the near future.

Finally, there is abundant literature on ChatGPT and mental health (Topic 4). Most of the academic literature on this topic focuses on the risks involved in mental health patients relying on ChatGPT. This was somewhat in contrast with the many tweets found on Topic 4 that highlighted the availability of ChatGPT as a provider of human-like interactions and personalized advice around the clock. The negative consequences discussed by the academicians and other professionals included escalation of self-isolation, which is known to lead to suicide or self-harm [43,44], risk of exposing sensitive personal information about themselves and their caregivers, which could lead to privacy violations [44,45], and ChatGPT’s failure in capturing nonverbal cues and subtle human signals and its tendency to underestimate the suicide risk [44,46]. The literature suggests that ChatGPT is particularly not equipped to serve younger generation and children who are more likely to rely on the LLM applications [44,47]. Also, ChatGPT in childcare can easily provide false information. The collection of data from children raises significant concerns regarding privacy, security and the risk of potential data misuse [48]. Furthermore, informed consent is a fundamental ethical requirement for AI-driven mental health apps, as outlined in WHO’s Regulatory Considerations on Artificial Intelligence for Health [49], which emphasizes the need for privacy and data protection, such as the General Data Protection Regulation (GDPR) in Europe and the Health Insurance Portability and Accountability Act (HIPAA).

In summary, we found that the general public is clearly privy to the main opinions raised by the academic community and health professionals, while the discrepancy in opinions was more notable in ChatGPT’s capability as a mental health service provider as well as its potential as an analytical tool. For both topics, the general public was somewhat more optimistic or less specific in providing negative opinions. However, it is important to note that academic literature, which provides a more authoritative and evidence-based perspective, holds greater significance and value than public opinion in evaluating these aspects. Public opinion, while informative, often lacks the depth and rigor that scholarly analysis offers in these domains.

This study acknowledges several limitations. First, tweets often express a mix of positive and negative sentiment and may also contain advertisements. This complexity can challenge both LLMs and human analysts in accurately classifying them. Future research could address this by developing methods to categorize each tweet based on percentages of positive and negative sentiment, and by training LLMs to predict the likelihood of advertisements. Second, the vast number of LLMs (over 10,000) makes it impractical to test them all within a single study. Future work could involve building a centralized platform that stores LLM parameters and facilitates the replication of research findings. Third, the previously free academic Twitter API for collecting census data is no longer available. This necessitates exploring paid alternatives for future studies involving census tweets. Fourth, besides X or Twitter, other social media platforms such as Facebook, Instagram, and Threads can also serve as valuable sources of public opinion. However, accessing data from these platforms presents significant challenges due to strict privacy policies and data access restrictions. Unlike X or Twitter, where public data is more accessible, these platforms impose restrictions that limit large-scale data collection and analysis. More resources are required to collect data and analyze such variations in pattern. Finally, studying X or Twitter users may not fully reflect general public opinion, as the characteristics of X or Twitter users may not be representative of the general population. X or Twitter users from different backgrounds may exhibit varying behaviors. Also, our data do not allow us to assess differences in sentiment and opinion between health care professionals and patients. A comparative study focusing on these differences would be valuable to capture a full spectrum of perspectives. Thus, the generalization of our results to the general population should be approached with caution.

### Conclusion

The public’s cautious optimism serves as a call to action for both technological developers and regulatory bodies to prioritize transparency, ethical standards, and the safeguarding of patient data as integral components of AI development in health care. Ensuring these measures should not only build public confidence but also enhance the efficacy and acceptance of AI in the health care sector overall. Further, divergent opinions across different healthcare AI topics indicate further research is warranted to better understand where AI can best add value without compromising ethical standards. Future studies should continue to track such public sentiment discussion and its correlation with real-world AI integration outcomes in healthcare. Over time, deeper insights into how public perceptions will evolve, effectively guiding successful LLM adoption in the health care space.

## Abbreviations

AI: artificial intelligence
GDPR: General Data Protection Regulation
HIPAA: Health Insurance Portability and Accountability Act
LLM: large language model
STM: structural topic modeling
